# Supplementary material for: Socioeconomic disparities and dog rabies: a retrospective analysis of high-spatial-resolution surveillance data from a Latin American city
Source: Lancet Reg Health Am. 2025 Oct 31;52:101285. doi: 10.1016/j.lana.2025.101285 (PMC12800993; doi:10.1016/j.lana.2025.101285)
Supplement: Summary Spanish Translation [file mmc2.pdf]

Editorial disclaimer: *This translation in Portuguese was submitted by the authors and we reproduce it as supplied. It has not been peer reviewed. Our editorial processes have only been applied to the original abstract in English, which should serve as reference for this manuscript.*

Desigualdades Socioeconómicas y Rabia Canina: Un Análisis Retrospectivo de Datos de Vigilancia de Alta Resolución Espacial en una Ciudad Latinoamericana

Sherrie Xie, PhD[1], Julianna Shinnick, MSN[1], Elvis W. Diaz, MVZ[2], Edith Zegarra, Lic.[3], Ynes Monroy, Lic.[3], Sergio E. Recuenco, DrPH[4], Ricardo Castillo-Neyra, PhD[1,2,5]

1. Departamento de Bioestadística, Epidemiología e Informática, Escuela de Medicina Perelman, Universidad de Pensilvania, Filadelfia, Pensilvania, EE. UU.
2. Laboratorio de Investigación en Enfermedades Zoonóticas, Facultad de Salud Pública y Administración, Universidad Peruana Cayetano Heredia, Lima, Perú
3. Laboratorio Regional de Referencia, Ministerio de Salud, Arequipa, Perú
4. Centro de Investigación en Tecnología, Biomedicina y Medio Ambiente (CITBM), Facultad de Medicina San Fernando, Universidad Nacional Mayor de San Marcos, Lima, Perú
5. Departamento de Patobiología, Facultad de Medicina Veterinaria, Universidad de Pensilvania, Filadelfia, Pensilvania, EE. UU.

RESUMEN

Antecedentes: La rabia humana transmitida por perros se asocia intuitivamente con la pobreza, pero pocos estudios han investigado formalmente la relación entre la desventaja socioeconómica a una escala local y la incidencia de rabia canina.

Métodos: Aprovechamos una base de datos de vigilancia única, con resolución espacial de alta precisión, de la ciudad de Arequipa, Perú, donde la rabia canina es endémica, para explorar la relación entre el nivel socioeconómico (NSE) del vecindario y el riesgo de rabia canina en 2015-2022. Los casos y las muestras de rabia se asignaron al nivel socioeconómico de su manzana o localidad de origen, respectivamente. Probamos la hipótesis de que un NSE más bajo se asocia con mayor positividad de casos y utilizamos un modelo estadístico espacial para entender cómo varió la positividad de muestras en el espacio.

Hallazgos: Los casos de rabia se concentraron en manzanas socioeconómicamente desfavorecidas ($p < 0{,}001$), y la positividad de muestras tuvo una asociación significativa y positiva con la desventaja socioeconómica del vecindario ($p < 0{,}05$ en todos los periodos), lo que sugiere que el esfuerzo de vigilancia fue bajo en relación con la incidencia de casos en áreas desfavorecidas. Estratificar las muestras según fueran recolectadas por vigilancia activa vs. pasiva reveló que la vigilancia activa redujo las desigualdades en el esfuerzo de vigilancia y en la positividad de muestras. El análisis espacial identificó un foco de positividad de muestras en una región socioeconómicamente desfavorecida con bajo acceso a instalaciones de salud.

Interpretación: Se sabe que la rabia transmitida por perros afecta a las regiones más pobres a nivel global. Encontramos patrones similares a una escala mucho más pequeña, dentro de los límites de una sola ciudad.

Un enfoque equilibrado que combine vigilancia espacialmente dirigida (“activa”) y comunitaria (“pasiva”) puede ayudar a reducir las desigualdades de rabia. Los programas de vacunación canina masiva y vigilancia podrían enfocarse en vecindarios desfavorecidos para asignar recursos a las áreas más afectadas y controlar de manera más eficaz las epidemias de rabia canina.
